# The VEGFR/PDGFR tyrosine kinase inhibitor, ABT-869, blocks necroptosis by targeting RIPK1 kinase

**DOI:** 10.1042/BCJ20230035

**Published:** 2023-05-12

**Authors:** Catia L. Pierotti, Annette V. Jacobsen, Christoph Grohmann, Ruby K. Dempsey, Nima Etemadi, Joanne M. Hildebrand, Cheree Fitzgibbon, Samuel N. Young, Katherine A. Davies, Wilhelmus J. A. Kersten, John Silke, Kym N. Lowes, Hélène Jousset Sabroux, David C. S. Huang, Mark F. van Delft, James M. Murphy, Guillaume Lessene

**Affiliations:** 1 The Walter and Eliza Hall Institute of Medical Research, Parkville, VIC 3052, Australia; 2 Department of Medical Biology, University of Melbourne, Parkville, VIC 3052, Australia; 3 Drug Discovery Biology, Monash Institute of Pharmaceutical Sciences, Monash University, Parkville, VIC 3052, Australia; 4 Department of Biochemistry and Therapeutics, University of Melbourne, Parkville, VIC 3052, Australia

**Keywords:** kinase inhibitor, necroptosis, protein-serine-threonine kinases, signalling

## Abstract

Necroptosis is a mode of programmed, lytic cell death that is executed by the mixed lineage kinase domain-like (MLKL) pseudokinase following activation by the upstream kinases, receptor-interacting serine/threonine protein kinase (RIPK)-1 and RIPK3. Dysregulated necroptosis has been implicated in the pathophysiology of many human diseases, including inflammatory and degenerative conditions, infectious diseases and cancers, provoking interest in pharmacological targeting of the pathway. To identify small molecules impacting on the necroptotic machinery, we performed a phenotypic screen using a mouse cell line expressing an MLKL mutant that kills cells in the absence of upstream death or pathogen detector receptor activation. This screen identified the vascular endothelial growth factor receptor (VEGFR) and platelet-derived growth factor receptor (PDGFR) tyrosine kinase inhibitor, ABT-869 (Linifanib), as a small molecule inhibitor of necroptosis. We applied a suite of cellular, biochemical and biophysical analyses to pinpoint the apical necroptotic kinase, RIPK1, as the target of ABT-869 inhibition. Our study adds to the repertoire of established protein kinase inhibitors that additionally target RIPK1 and raises the prospect that serendipitous targeting of necroptosis signalling may contribute to their clinical efficacy in some settings.

## Introduction

Necroptosis is a caspase-independent mode of regulated cell death that is morphologically characterised by cell swelling, plasma membrane rupture, and the spillage of the cellular contents into the extracellular milieu initiating an inflammatory response [1–3]. Ancestrally, necroptosis is thought to have evolved as an altruistic host defence pathway, which is reflected by convergent evolution of analogous pathways in plants and fungi [4–6], and the discovery of pathogen-encoded protein inhibitors of the pathway [7–10]. Contemporary interest in the pathway has been driven by the implication of errant necroptosis in the pathophysiology of inflammatory diseases [11], such as those of the skin [12], gut [13], kidney [14,15] and lung [16], which has prompted much interest in targeting the pathway therapeutically.

The necroptosis pathway is triggered by activation of death receptors, such as tumour necrosis factor (TNF) receptor 1, or pathogen detectors, including Toll-like receptor (TLR)-3, TLR4 and Z-DNA binding protein 1 (ZBP1) [17]. In circumstances when the cellular inhibitor of apoptosis protein (cIAP) E3 ubiquitin ligase family and the pro-apoptotic proteolytic enzyme, Caspase-8, are depleted or inactivated, necroptosis can proceed. Autophosphorylation of the apical necroptotic kinase, RIPK1, prompts assembly of a high molecular weight cytoplasmic complex termed the necrosome, which is nucleated by the hetero-oligomerisation of RIPK1 and the downstream necroptosis effector kinase, RIPK3 [18]. Subsequently, the terminal effector of the pathway, the mixed lineage kinase domain-like (MLKL) pseudokinase, is recruited to the necrosome [19] and phosphorylated by RIPK3 [20–22] to trigger MLKL dissociation, interconversion into an active conformer [23], oligomerisation and translocation to the plasma membrane [24–26]. At the membrane, MLKL oligomers assemble into hotspots [26,27] and the N-terminal four-helix bundle domain of MLKL disrupts the membrane to induce cell swelling, lysis and expulsion of immunogenic cellular constituents into the extracellular milieu [28–33].

While necroptosis signalling is tightly regulated in human cells [25,34,35], our previous studies revealed that mutations in the pseudokinase domain can convert mouse MLKL into a constitutively lethal form [20]. We observed that expression of mouse MLKL harbouring mutations to emulate RIPK3-mediated phosphorylation of the MLKL pseudokinase domain activation loop, or of adjacent residues in the ATP-binding site, leads to constitutive cell death upon expression alone, in the absence of upstream necroptotic stimuli [20]. Using one of these constitutively activated mouse MLKL mutants — Q343A — expressed in mouse dermal fibroblasts (MDFs), we established a cell-based phenotypic screen to identify small molecules that modulate necroptosis [36,37]. This approach previously identified two inhibitors of necroptosis, 17-AAG [36] and AMG-47a [37], which target the chaperone, HSP90, and the kinases, RIPK1 and RIPK3, respectively. Here, we identify ABT-869 — a small molecule previously described as an inhibitor of tyrosine kinases of the VEGF and PDGF receptor families [38] — as a novel inhibitor of necroptosis. Using biochemical, biophysical and cellular studies, we characterise the apical necroptotic kinase, RIPK1, as a previously unrecognised target of ABT-869. In concert with other reports, our findings raise the possibility that off-target inhibition of necroptotic signalling by established kinase inhibitors may contribute to their therapeutic efficacy in some contexts.

## Results

### ABT-869 is a previously unreported inhibitor of necroptosis

**Figure 1.**
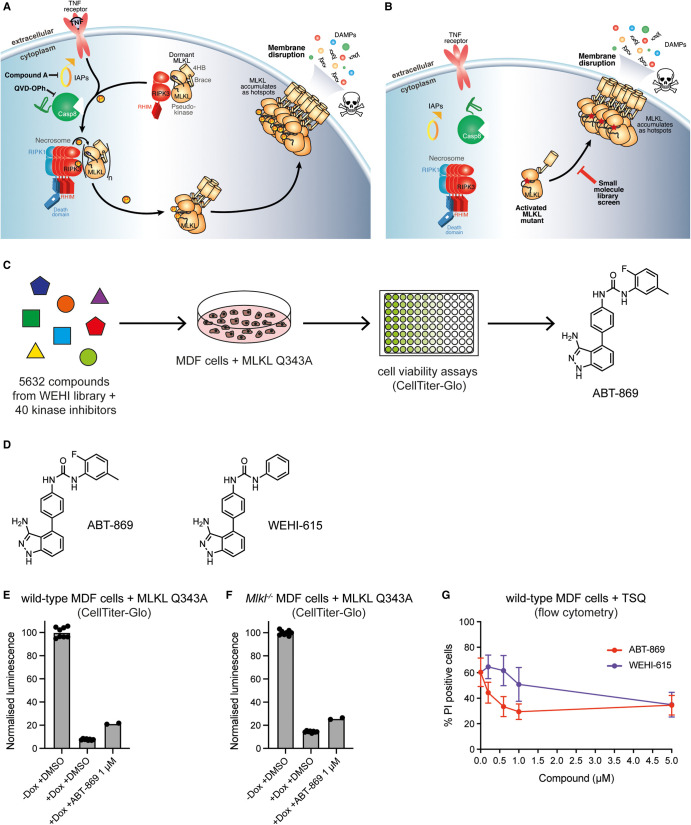
ABT-869 is a previously unreported inhibitor of necroptosis. (**A**) Schematic of the necroptosis pathway. TNF (T) activates TNFR1, the Smac-mimetic Compound A (S) blocks cIAP activity and the pan-caspase inhibitor Q-VD-OPh (Q) blocks caspase-8 activity. This TSQ stimulus results in activation of RIPK1 and RIPK3, and subsequent phosphorylation and activation of MLKL, which causes MLKL-mediated membrane disruption and cell death. (**B**) Schematic of the constitutively activated mouse MLKL mutant, Q343A. Expression of MLKL Q343A using doxycycline causes cell death in the absence of upstream necroptotic stimuli. This enabled a cell-based phenotypic screen for small molecules that modulate necroptosis at the level or downstream of MLKL activation. The skull and crossbones image (Mycomorphbox_Deadly.png; by Sven Manguard) in (**A**,**B**) was used under a Creative Commons Attribution-Share Alike 4.0 license. (**C**) Schematic of the cell-based phenotypic screen. A total of 5632 compounds from the WEHI small molecule library along with 40 kinase inhibitors were screened against wild-type or *Mlkl^−/−^* mouse dermal fibroblast (MDF) cells expressing the MLKL Q343A mutant. The ability of the small molecules to inhibit cell death was measured by CellTiter-Glo cell viability assays. ABT-869, a VEGF and PDGF receptor tyrosine kinase inhibitor, was identified as a hit. See also Supplementary Figure S1A. (**D**) Chemical structure of ABT-869 and its analogue WEHI-615. (**E**) Wild-type mouse dermal fibroblast (MDF) cells expressing the doxycycline-inducible MLKL Q343A mutant to trigger constitutive necroptosis were treated with DMSO alone, doxycycline (Dox; 1 µg/ml) and DMSO, or Dox and ABT-869 (1 µM). Cell viability was quantified by CellTiter-Glo. Data represent the mean of ≥2 technical replicates from a single experiment, with individual data points shown. See also Supplementary Figure S1A. (**F**) *Mlkl^−/−^* mouse dermal fibroblast (MDF) cells expressing the doxycycline-inducible MLKL Q343A mutant to trigger constitutive necroptosis were treated with DMSO alone, doxycycline (Dox; 1 µg/ml) and DMSO, or Dox and ABT-869 (1 µM). Cell viability was quantified by CellTiter-Glo. Data represent the mean of ≥2 technical replicates from a single experiment, with individual data points shown. See also Supplementary Figure S1A. (**G**) Wild-type mouse dermal fibroblast (MDF) cells were stimulated with TSQ (TNF, Smac-mimetic, Q-VD-OPh) to induce necroptosis and treated with increasing concentrations of ABT-869 or WEHI-615. Cell death was quantified by propidium iodide (PI) staining using flow cytometry. Data represent the mean of *n* = 4 independent experiments and errors bars represent SEM.

To identify small molecules that modulate necroptosis at the level of MLKL or downstream of MLKL in the pathway, we performed a cell-based phenotypic screen using MDF cells expressing the MLKL Q343A mutant. In wild-type cells, necroptosis can be induced with the TSQ stimulus (TNF, T; Smac-mimetic Compound A, S; and pan-Caspase inhibitor Q-VD-OPh, Q) (Figure 1A). In cells where an exogene encoding the MLKL Q343A mutant is inducibly expressed using doxycycline, MLKL Q343A triggers cell death in the absence of additional necroptotic stimulation (Figure 1B) [36,37]. With this focused approach, a random selection of 5632 compounds from a diverse pharmacophore, in-house small molecule library and an additional 40 known kinase inhibitors available in-house were screened for their ability to inhibit cell death induced by the expression of this MLKL construct (Figure 1C). The suppression of cell death by these inhibitors was monitored using CellTiter-Glo cell viability assays to enable high-throughput screening. From this screen, a small molecule within the kinase inhibitor subset, ABT-869 (Figure 1D; Supplementary Figure S1A), was identified as an inhibitor of MLKL Q343A-induced death. ABT-869 was originally described as a multi-targeted receptor tyrosine kinase inhibitor developed by AbbVie that targets all members of the VEGF and PDGF receptor tyrosine kinase families [39]. ABT-869 attenuated necroptosis induced by expression of the MLKL Q343A constitutively active mutant in wild-type or *Mlkl^−/−^* MDF cells when applied at 1 µM concentration (Figure 1E,F).

To validate ABT-869 as a hit from the screen, we performed cellular studies, which demonstrated that ABT-869 also inhibited necroptosis induced by TSQ treatment in wild-type MDF cells and displayed concentration-dependent inhibition, as monitored by propidium iodide (PI) uptake using flow cytometry (Figure 1G). PI uptake provides a direct method of quantifying death at the level of each cell in a population, while CellTiter-Glo only offers a population-level assessment of viability. An analogue of ABT-869, WEHI-615 (Figure 1D), lacking the terminal ring fluorine and methyl substituents, was subsequently synthesised to evaluate the specificity of ABT-869 as a necroptosis inhibitor. This analogue conferred less potent inhibition of TSQ-induced necroptosis than ABT-869 in wild-type MDF cells (Figure 1G).

**Figure 2.**
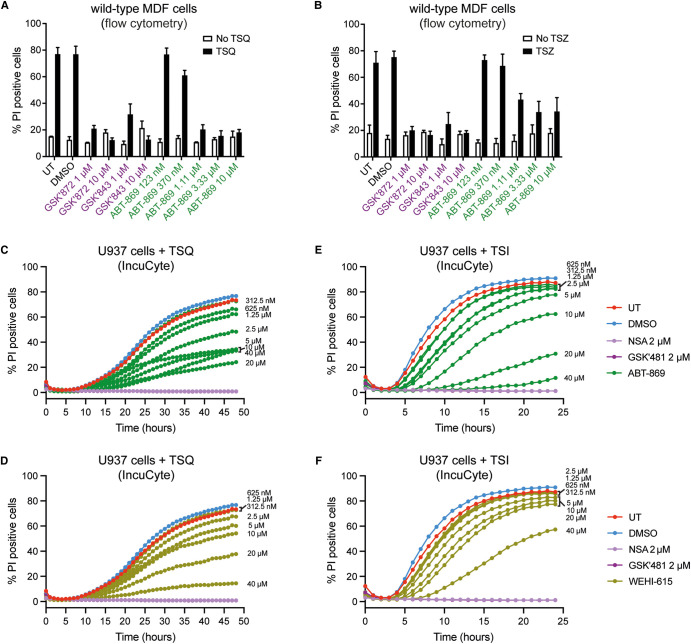
ABT-869 inhibits necroptosis in mouse and human cells. (**A**,**B**) Wild-type mouse dermal fibroblast (MDF) cells were treated with increasing concentrations of ABT-869 or control compounds, RIPK3 inhibitors GSK′872 and GSK′843, DMSO alone or left untreated (UT) for 1 h then stimulated with TSQ (TNF, Smac-mimetic, Q-VD-OPh) (**A**) or TSZ (TNF, Smac-mimetic, z-VAD-fmk) (**B**) for 24 h to induce necroptosis. Cell death was quantified by propidium iodide (PI) staining using flow cytometry. Data represent the mean of *n* = 3 (**A**) or *n* = 4 (**B**) independent experiments and error bars represent SEM. (**C**–**F**) Human U937 cells were treated with increasing concentrations of ABT-869 or control compounds, MLKL inhibitor NSA and RIPK1 inhibitor GSK′481, DMSO alone or left untreated (UT) for 1 h then stimulated with TSQ (TNF, Smac-mimetic, Q-VD-OPh) for 48 h (**C**) or TSI (TNF, Smac-mimetic, IDN-6556) for 24 h (**E**) to induce necroptosis. Parallel experiments were performed to assess protection of TSQ (**D**) or TSI (**F**) induced death in the presence of the ABT-869 analogue, WEHI-615. Cell death was monitored by SPY505 (live cells) and propidium iodide (PI; dead cells) uptake using IncuCyte live cell imaging. One representative result shown from *n* = 4 (**C**,**D**) or *n* = 3 (**E**,**F**) independent experiments. See also Supplementary Figure S2A–H.

We next performed a 5-point 3-fold titration from 10 µM of ABT-869 in wild-type MDF cells following stimulation with either TSQ (Figure 2A) or another necroptotic stimulus, TSZ, comprising TNF (T), Smac-mimetic Compound A (S) and the pan-Caspase inhibitor z-VAD-fmk (Z) (Figure 2B). ABT-869 showed concentration-dependent inhibition of necroptotic cell death triggered by either TSQ or TSZ, as measured by PI uptake using flow cytometry. Interestingly, the protection was comparable between ABT-869 and two control necroptosis inhibitors, GSK′872 and GSK′843 (Supplementary Figure S1B) that target RIPK3 [40], at concentrations >1 µM when wild-type MDF cell death was induced with TSQ treatment (Figure 2A). In contrast, when these cells were treated with TSZ, a more potent death stimulus than TSQ [41,42], we observed more potent protection with the control inhibitors compared with ABT-869 (Figure 2B).

### ABT-869 inhibits necroptosis in mouse and human cells

As ABT-869 was identified in a screen that used mouse cells expressing a constitutively active MLKL mutant and as our validation studies were performed in mouse cell lines, we further profiled the activity of ABT-869 and WEHI-615 in human cells. In U937 cells, a human lymphoma cell line commonly used to study necroptosis, we performed an 8-point 2-fold titration from 40 µM of ABT-869 and WEHI-615 where cell death was measured by PI uptake using IncuCyte live cell imaging following necroptosis induction. We used IncuCyte imaging to quantify cell death because this method allowed us to monitor the kinetics of cell death (PI uptake) over time. In contrast, using flow cytometry to quantify death by PI uptake only offers a snapshot of cell death at a fixed timepoint. ABT-869 blocked cell death when necroptosis was triggered with TSQ in U937 cells, and although variability between experiments made it difficult to accurately determine an IC_50_, protection from cell death was observed at concentrations >1.25 µM (Figure 2C; Supplementary Figure S2E). Similar to what was observed in MDF cells, WEHI-615 more weakly impaired necroptosis in TSQ-stimulated U937 cells, with an IC_50_ of 35 µM (Figure 2D; Supplementary Figure S2F). TSI, comprising TS and the pan-Caspase inhibitor IDN-6556/Emricasan (I), is a more potent necroptosis trigger than TSQ or TSZ [35,41]. When U937 cells were stimulated with TSI, ABT-869 showed reduced inhibition of necroptosis, compared with TSQ stimulation, with an IC_50_ of 22 µM (Figure 2E; Supplementary Figure S2G). WEHI-615 only showed marked attenuation of TSI-stimulated cell death at the highest concentration tested (40 µM) (Figure 2F; Supplementary Figure S2H). Neither ABT-869 nor WEHI-615 showed any marked toxicity when applied to U937 cells in the absence of TSQ or TSI (Supplementary Figure S2A–D). Together, these data validate ABT-869 and its less active analogue WEHI-615 as previously unreported inhibitors of necroptosis in mouse and human cells.

### ABT-869 binds to RIPK1, but not to RIPK3 or MLKL, *in vitro* and in cells

Previously, we identified two necroptosis inhibitors using a similar screening strategy: the kinase inhibitor, AMG-47a, which directly targets RIPK1 and RIPK3 [37], and the HSP90 chaperone protein inhibitor, 17-AAG, which indirectly influences the folding of the HSP90 client proteins, RIPK1, RIPK3 and MLKL [36]. In light of these findings, and given that the core machinery required for TNF-dependent necroptosis comprises of two kinases (RIPK1, RIPK3) and a pseudokinase (MLKL) [27], we sought to establish if ABT-869, a kinase inhibitor, could also target these proteins to inhibit necroptosis.

**Figure 3.**
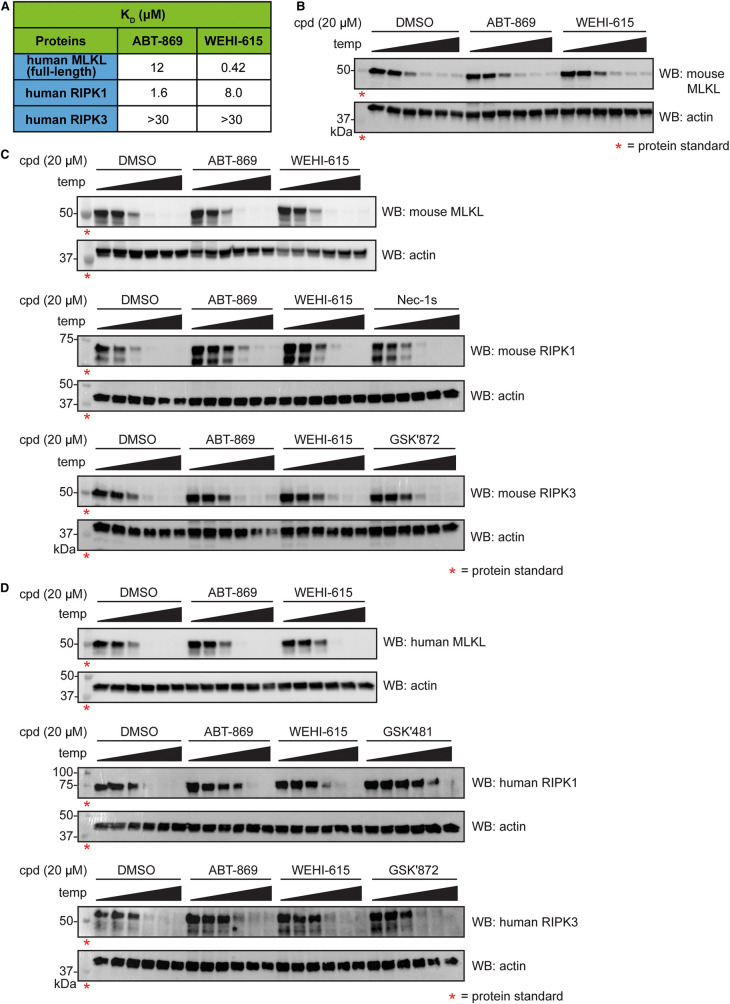
ABT-869 binds to RIPK1 in mouse and human cells. (**A**) Binding affinities (*K*_D_) of ABT-869 and WEHI-615 for human full-length MLKL, RIPK1 kinase domain and RIPK3 kinase domain measured by competition binding assays from the DiscoverX KINOME*scan* platform using the *Kd*ELECT service. Each value is the mean of two replicates. (**B**–**D**) Cellular Thermal Shift Assays (CETSA) in mouse and human cells. *Mlkl^−/−^* mouse dermal fibroblast (MDF) cells expressing MLKL Q343A (**B**), wild-type MDF cells (**C**) and human U937 cells (**D**) were treated with DMSO, ABT-869, WEHI-615, RIPK1 inhibitor Nec-1s, RIPK3 inhibitor GSK′872 or human RIPK1 inhibitor GSK′481 (all 20 µM). Cells were subjected to an increasing temperature gradient focused around the melting temperature of the protein of interest. Following the separation of soluble and insoluble proteins, the remaining soluble proteins were detected by Western blot. Red asterisks denote protein standards. One representative result shown from *n* = 3 (**B**,**C**) or *n* = 2–3 (**D**) independent experiments. See also Supplementary Figure S3A–C.

We first assessed the ability of ABT-869 and WEHI-615 to bind the necroptotic effectors *in vitro* using competition binding assays (DiscoverX KINOME*scan* platform) (Figure 3A), where compound binding to a given kinase or pseudokinase is determined through competition with an ATP-site directed probe [43]. ABT-869 and WEHI-615 bound to the human RIPK1 kinase domain with a *K*_D_ of 1.6 µM and 8.0 µM, respectively. Neither compound bound the human RIPK3 kinase domain at any concentration tested (up to 30 µM). Interestingly, ABT-869 showed weak binding to full-length human MLKL with a *K*_D_ of 12 µM, whereas WEHI-615 displayed stronger binding with a *K*_D_ of 0.42 µM. As the IC_50_ for ABT-869 inhibition of necroptotic cell death is estimated to be ∼5 µM in TSQ-treated U937 cells compared with the IC_50_ of 35 µM for WEHI-615, and the ABT-869 binding affinity for RIPK1 is >7-fold higher than for MLKL, we hypothesised that targeting RIPK1, rather than MLKL, is likely to be the mechanism by which ABT-869 blocks cell death.

Next, we wanted to establish if ABT-869 and WEHI-615 interact with the necroptotic effector proteins in a cellular context. Using Cellular Thermal Shift Assays (CETSA) to evaluate cellular target engagement [44], we found that neither ABT-869 nor WEHI-615 had any effect on the thermal stability of the MLKL Q343A mutant expressed in *Mlkl^−/−^* MDF cells (Figure 3B; Supplementary Figure S3A). Furthermore, neither compound interacted with endogenous mouse MLKL in MDF cells or human MLKL in U937 cells (Figure 3C,D; Supplementary Figure S3B,C). In contrast, both ABT-869 and WEHI-615 increased the thermal stability of endogenous mouse RIPK1 in MDF cells, similar to the established RIPK1 inhibitor, Nec-1s (Supplementary Figure S1B) [45,46]. Both ABT-869 and WEHI-615 also increased the thermal stability of endogenous human RIPK1 in U937 cells, albeit not as profoundly as the established human RIPK1 inhibitor, GSK′481 (Supplementary Figure S1B) [47]. Neither ABT-869 nor WEHI-615 impacted the thermal stability of endogenous mouse RIPK3 in MDF cells or human RIPK3 in U937 cells. Interestingly, the established RIPK3 kinase inhibitor, GSK′872 [40], also had no effect on RIPK3 stability in MDF or U937 cells, consistent with previously published findings [37,48]. However, given that GSK′872 detectably binds recombinant RIPK3 kinase domain *in vitro*, while ABT-869 and WEHI-615 did not, it is likely that neither ABT-869 nor WEHI-615 bind to RIPK3 in cells. As in some cases compound binding fails to induce substantial change in the thermal stability of the target protein [44], we hypothesise this is the case for GSK′872 with RIPK3. Overall, the CETSA data suggest that ABT-869 interacts with RIPK1, but not RIPK3 or MLKL, in both mouse and human cells.

**Figure 4.**
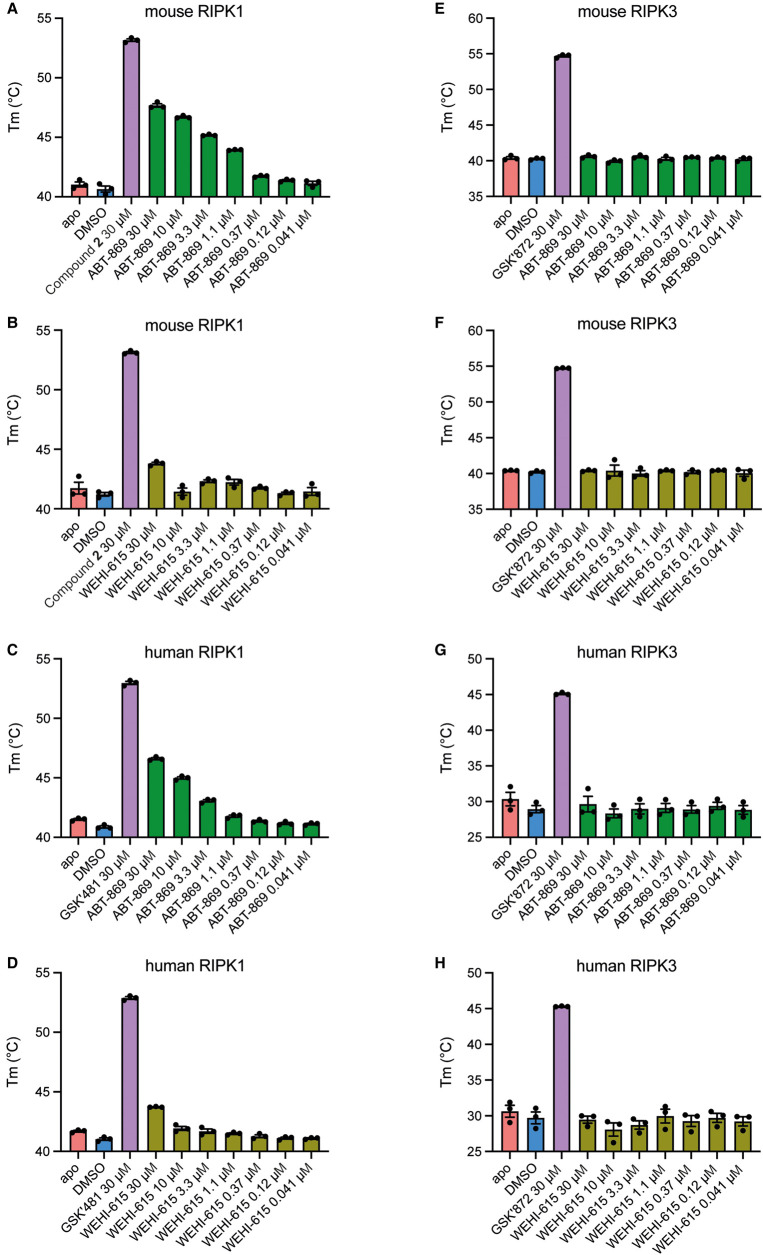
ABT-869 binds to mouse and human RIPK1 *in vitro*. Thermal Shift Assays (TSA) with mouse and human RIPK1 and RIPK3 kinase domains. Increasing concentrations of ABT-869 or WEHI-615 were tested for their ability to alter the melting temperature (*T*_M_) of mouse RIPK1 (9.5 µg) (**A**,**B**), human RIPK1 (12 µg) (**C**,**D**), mouse RIPK3 (10 µg) (**E**,**F**) and human RIPK3 (6.5 µg) (**G**,**H**) compared with the positive controls Compound 2 [48] for mouse RIPK1, GSK′481 for human RIPK1 and GSK′872 for mouse and human RIPK3 (all 30 µM). Data represent the mean of *n* = 3 independent experiments and error bars represent SEM. See also Supplementary Figure S4A–H.

We then used Thermal Shift Assays (TSA) to further investigate the binding of ABT-869 and WEHI-615 to RIPK1 *in vitro*. In TSA, changes in the thermal stability of the target protein can be monitored using a fluorescent dye that binds hydrophobic residues, which become exposed as the protein unfolds upon increasing temperature [49]. Increasing concentrations of ABT-869 resulted in an increase in the melting temperature (*T*_M_) of recombinant mouse RIPK1 kinase domain, with a *K*_D_ of 1.63 µM obtained for this binding interaction (Figure 4A; Supplementary Figure S4A). WEHI-615 only increased the *T*_M_ of mouse RIPK1 at the highest concentration tested (30 µM) (Figure 4B; Supplementary Figure S4B). Similarly, ABT-869 increased the *T*_M_ of recombinant human RIPK1 kinase domain, with a *K*_D_ of 7.33 µM, while WEHI-615 only increased the *T*_M_ of human RIPK1 at the highest concentration tested (30 µM) (Figure 4C,D; Supplementary Figure S4C,D). Neither ABT-869 nor WEHI-615 had any effect on the *T*_M_ of recombinant mouse or human RIPK3 kinase domain (Figure 4E–H; Supplementary Figure S4E–H). The findings using recombinant proteins in TSA and DiscoverX binding experiments mirror the trends observed in the CETSA experiments, and collectively implicate RIPK1 as the target through which ABT-869 blocks necroptosis.

### ABT-869 inhibits RIPK1 kinase activity *in vitro* and in cells

**Figure 5.**
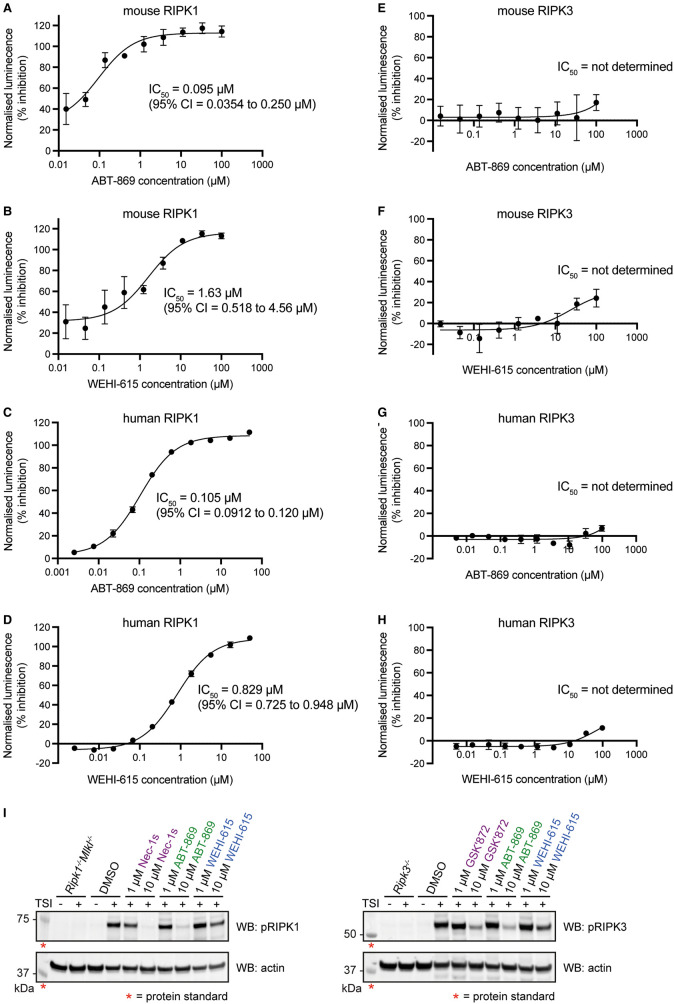
ABT-869 inhibits RIPK1 kinase activity *in vitro* and in cells. (**A**–**H**) *In vitro* phosphorylation assays with mouse and human RIPK1 and RIPK3 kinase domains measured by ADP-Glo Kinase Assays. Increasing concentrations of ABT-869 or WEHI-615 were tested for their ability to inhibit the autophosphorylation (IC_50_) of mouse RIPK1 (200 nM) (**A**,**B**), human RIPK1 (200 nM) (**C**,**D**), mouse RIPK3 (10 nM) (**E**,**F**) and human RIPK3 (10 nM) (**G**,**H**). Data represent the mean of *n* = 3 (**A**,**B**,**E**,**F**) or *n* = 2 (**C**,**D**,**G**,**H**) independent experiments and error bars represent SEM. (**I**) Cellular phosphorylation assays. Wild-type mouse dermal fibroblast (MDF) cells were treated with DMSO, ABT-869, WEHI-615, RIPK1 inhibitor Nec-1s or RIPK3 inhibitor GSK′872 for 2 h then stimulated with TSI (TNF, Smac-mimetic, IDN-6556) for 2 h to induce autophosphorylation of RIPK1 and RIPK3. *Ripk1^−/−^Mlkl^−/−^* MDF cells and *Ripk3^−/−^* MDF cells were included as controls. Phospho-RIPK1 and phospho-RIPK3 protein levels were detected from whole cell lysates by Western blot. Red asterisks denote protein standards. One representative result shown from *n* = 3 independent experiments. See also Supplementary Figure S5A–C.

To determine whether the binding of ABT-869 to RIPK1 impacts its kinase activity, we performed ADP-Glo Kinase Assays to evaluate RIPK1 autophosphorylation activity *in vitro* in the presence of ABT-869 or WEHI-615. Increasing concentrations of ABT-869 resulted in increasing inhibition of recombinant mouse RIPK1 kinase activity, with an IC_50_ of 95 nM (Figure 5A). WEHI-615 inhibited mouse RIPK1 kinase activity less potently, with an IC_50_ of 1.6 µM (Figure 5B). A similar trend was observed for human RIPK1, where ABT-869 inhibited recombinant human RIPK1 kinase activity with an IC_50_ of 105 nM (Figure 5C) and WEHI-615 with an IC_50_ of 829 nM (Figure 5D). In addition, neither compound inhibited recombinant mouse or human RIPK3 kinase activity, aside from some low level inhibition at the highest concentration tested (100 µM) (Figure 5E–H). These results mirror the trends observed in the TSA binding data, and demonstrate that ABT-869 binds to and inhibits RIPK1, but not RIPK3, *in vitro*.

We then assessed the influence of ABT-869 on RIPK1 kinase activity in cells. TNF-driven necroptosis induction results in the autophosphorylation of RIPK1 and RIPK3, which are key events in this cell death signalling pathway [22,50–55]. Initially, we performed time course experiments to map the chronology of mouse RIPK1 and RIPK3 autophosphorylation events in MDF cells over a 4 h period following necroptosis induction (Supplementary Figure S5A,B). By Western blot, we detected autophosphorylation of S166 in the RIPK1 activation loop at 1 h, which was maintained at 2 h and decreased at 3 and 4 h. Autophosphorylation of the RIPK3 C-lobe residues, T231/S232, was observed at 2 h, with levels maintained at 3 h and 4 h. Total RIPK1 and RIPK3 levels remained constant over this 4 h time period following TSI stimulation.

As maximal RIPK1 autophosphorylation was observed 2 h post-TSI stimulation, MDF cells treated with ABT-869, WEHI-615 and control inhibitors were stimulated with TSI for 2 h and phospho-protein levels were determined by Western blot (Figure 5I; Supplementary Figure S5C). ABT-869 at 10 µM almost completely ablated RIPK1 phosphorylation, to a similar extent as the control RIPK1 inhibitor, Nec-1s, whereas WEHI-615 only partially reduced the phospho-RIPK1 signal at 10 µM. Similarly, the phospho-RIPK3 signal observed following 2 h TSI stimulation was almost completely inhibited by 10 µM ABT-869, to a similar extent to the control RIPK3 kinase inhibitor, GSK′872, while 10 µM WEHI-615 only partially reduced RIPK3 phosphorylation. However, given that ABT-869 did not bind to RIPK3 *in vitro* or in cells and did not inhibit RIPK3 kinase activity *in vitro*, the observed inhibition of RIPK3 autophosphorylation in cells is most likely attributable to the upstream inhibition of RIPK1 by ABT-869, which would prevent the activation and autophosphorylation of RIPK3. Together, these data demonstrate that ABT-869 inhibits RIPK1 kinase activity *in vitro* and in cells.

## Discussion

In a cell-based phenotypic screen for small molecules that block the killing mediated by an activated form of the necroptotic executioner, MLKL, we identified the kinase inhibitor, ABT-869 (Linifanib). Although ABT-869 was developed as a receptor tyrosine kinase inhibitor, here we identified the RIPK1 kinase as the target underpinning the ability of ABT-869 to inhibit necroptosis. ABT-869 blocked necroptotic signalling in mouse and human cell lines treated with a necroptotic stimulus cocktail (TSQ, TSZ or TSI), and death induced upon expression of an activated mutant form of mouse MLKL. RIPK1 was validated as the target of ABT-869 in cells using CETSA and immunoblots for necroptosis effector phosphorylation, in DiscoverX KINOME*scan* competition assays, and thermal shift and enzymatic assays using recombinant proteins. Consistent with our findings, Yu et al. [56] in a very recent parallel study, validated ABT-869 as an inhibitor of the necroptosis pathway, which could target RIPK1 to confer protection in mice from TNF-induced sterile sepsis.

**Figure 6.**
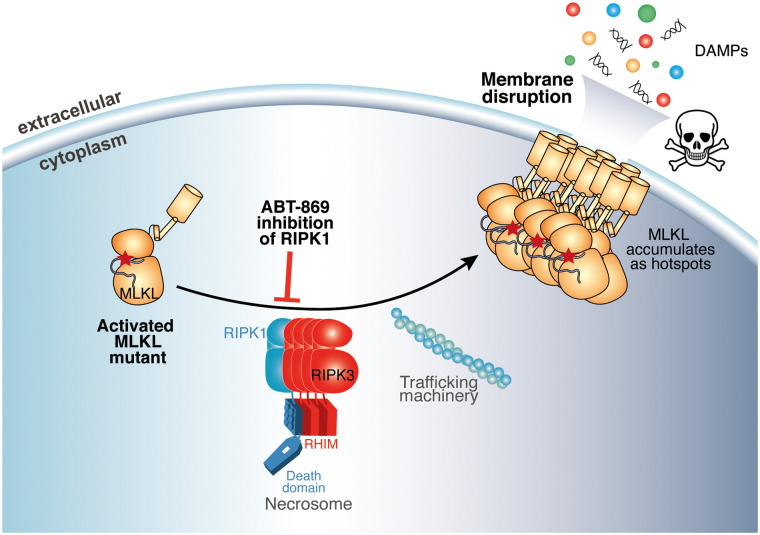
ABT-869 targets RIPK1 to block necroptosis. Schematic of the ABT-869 mechanism of action in the context of necroptosis inhibition. Necroptosis mediated by the constitutively activated MLKL Q343A mutant was inhibited by ABT-869, which binds to and inhibits RIPK1, implicating RIPK1 as having a role downstream of MLKL activation in the signalling pathway. The skull and crossbones image (Mycomorphbox_Deadly.png; by Sven Manguard) was used under a Creative Commons Attribution-Share Alike 4.0 license.

RIPK1 is typically considered to be the apical kinase in the necroptosis pathway, which acts upstream of RIPK3 and MLKL in directing cell death, following exposure to a death receptor ligand or pathogen molecular pattern. Recent data, however, have implicated RIPK1 as serving an important function downstream of MLKL activation [37], likely in scaffolding the assembly of the high molecular weight cytoplasmic platform termed the necrosome. Our findings provide further support for this idea, because cell death mediated by a constitutively activated form of MLKL could be inhibited by ABT-869, which targets RIPK1, but not RIPK3 or MLKL, implicating RIPK1 as performing a function in signalling subsequent to MLKL activation (Figure 6).

Our data add to a growing repertoire of tyrosine kinase inhibitors that target RIPK1 to block necroptosis via off-target mechanisms. Recently, an FGF receptor inhibitor, AZD4547, was reported to potently inhibit necroptosis by targeting RIPK1 [57]. This adds to several other tyrosine kinase inhibitors (reviewed in [58]), which includes sunitinib [59], pazopanib [59], ponatinib [60], and AMG-47a [37], now known to bind and inhibit RIPK1 in an off-target manner. Broadly speaking, this raises the prospect that other clinically approved kinase inhibitors may also act synergistically on the necroptosis pathway. The extent to which this binding may ameliorate inflammatory signalling by dampening necroptotic cell death and thus impact the clinical efficacy remains of outstanding interest.

Our findings underscore the challenges associated with identifying compounds that interfere with the terminal steps in necroptosis signalling using cell-based screens. RIPK1 appears to play two critical roles in the necroptosis pathway: (i) upon autophosphorylation, RIPK1 scaffolds the assembly of the necrosome with RIPK3; and (ii) RIPK1-scaffolded necrosome assembly is required to connect activated MLKL to the trafficking machinery to enable plasma membrane translocation and lytic cell death. These upstream and downstream signalling roles of RIPK1 in the necroptosis pathway rationalise the prevalence of RIPK1 inhibitors identified in cell-based screens as pathway inhibitors. Similar approaches have successfully identified inhibitors that target RIPK1 and RIPK3 kinase activity [37], and the HSP90 chaperone [36], which is critical to RIPK1, RIPK3 and MLKL stability, in cell-based screens. Future efforts will be important to identify inhibitors that target the terminal steps in the pathway at the level of MLKL oligomerisation and/or membrane association, or downstream at the level of as-yet-unknown (co)effectors.

## Materials and methods

### Compounds

ABT-869 and WEHI-615 were synthesised as previously reported [39]. GSK′872 [40], GSK′843 [40] and GSK′481 [47] were kindly supplied by Catalyst Therapeutics. Compound 2 was synthesised as previously described [48]. Nec-1s [45,46] was purchased from Sigma–Aldrich and NSA [21] was purchased from Merck Millipore.

### Reagents

**Table:** 

Necroptosis-Inducing Stimuli			
Stimulus	Component	Supplier	Concentration
TSQ	TNF	Produced in-house [61]	100 ng/ml
	Smac mimetic (Compound A)	Tetralogic	500 nM
	Q-VD-OPh	MP Biologicals	10 µM
TSZ	TNF	Produced in-house	100 ng/ml
	Smac mimetic (Compound A)	Tetralogic	500 nM
	z-VAD-fmk	ApexBio	10 µM
TSI	TNF	Produced in-house	100 ng/ml
	Smac mimetic (Compound A)	Tetralogic	500 nM
	IDN-6556 (Emricasan)	Tetralogic	5 µM

**Table:** 

Western Blot Primary Antibodies				
Protein	Antibody	Supplier	Catalogue #	Dilution
Mouse MLKL	Rat anti-MLKL clone 3H1	Produced in-house [20], available from Merck Millipore	cat #MABC604	1 : 1000
Mouse RIPK1	Rabbit anti-RIPK1	Cell Signalling Technology	cat #3493	1 : 1000
Mouse RIPK3 (CETSA)	Rabbit anti-RIPK3	ProSci	cat #2283	1 : 1000
Mouse RIPK3 (phosphorylation time course)	Rat anti-mouse RIPK3 clone 8G7	Produced in-house [9], available from Merck Millipore	cat #MABC1595	1 : 1000
Human MLKL	Rat anti-MLKL clone 3H1	Produced in-house [20], available from Merck Millipore	cat #MABC604	1 : 1000
Human RIPK1	Mouse anti-RIPK1	BD Transduction Laboratories	cat #610458	1 : 1000
Human RIPK3	Rat anti-human RIPK3 clone 1H2	Produced in-house [9], available from Merck Millipore	cat #MABC1640	1 : 1000
Phospho-mouse MLKL	Rabbit anti-mouse MLKL pS345	Abcam	cat #ab196436	1 : 1000
Phospho-mouse RIPK1	Rabbit anti-mouse RIPK1 pS166	Cell Signaling Technology	cat #31122	1 : 1000
Phospho-mouse RIPK3	Rabbit anti-mouse RIPK3 pT231/pS232	Kindly supplied by Genentech, GEN135-35-9 [62]	N/A	1 : 5000
Actin	Chicken anti-β-actin-HRP	Santa Cruz Biotechnology	cat #sc-47778 HRP	1 : 5000

**Table:** 

Western Blot Secondary Antibodies			
Antibody	Supplier	Catalogue #	Dilution
Goat anti-rat-HRP	Southern Biotech	cat #3010-05	1 : 5000 or 1 : 10 000
Goat anti-mouse-HRP	Southern Biotech	cat #1010-05	1 : 5000 or 1 : 10 000
Goat anti-rabbit-HRP	Southern Biotech	cat #4010-05	1 : 5000 or 1 : 10 000

### Western blotting general procedure

Proteins were transferred to nitrocellulose membranes using the iBlot Dry Blotting System (Invitrogen). Membranes were blocked with 5% w/v skim milk in PBS-T (PBS + 0.1% v/v Tween-20) for 1 h at room temperature, washed 3 × 20 min with PBS-T at room temperature, probed overnight at 4°C with the primary antibody for the protein of interest in PBS + 2% w/v BSA + 0.02% w/v NaN_3_, washed as above, probed for 1 h at room temperature with the relevant secondary antibody in PBS-T + 5% w/v BSA and washed as above. Immobilon Forte Western HRP Substrate (Merck Millipore) or Pierce ECL Western Blotting Substrate (Thermo Scientific) was added to the membranes and Western blots were developed using chemiluminescence on the ChemiDoc Imaging System (Bio-Rad). Membranes were then washed overnight with PBS-T, reprobed with the loading control (actin-HRP) for 1 h at room temperature, washed 3 × 20 min with PBS-T at room temperature and developed as described above.

### Cell culture and cell lines

MDF cells were cultured in DMEM (in-house). U937 cells were cultured in human tonicity RPMI (in-house). All media contained penicillin and streptomycin (100× solution, 10 000–12 000 units/ml penicillin and 10–12 mg/ml streptomycin in 0.9% w/v NaCl; Sigma–Aldrich) and was supplemented with 8–10% v/v Fetal Calf Serum (FCS; GE Healthcare, cat #SH30084.03). Wild-type (WT), *Mlkl^−/−^* expressing the MLKL Q343A mutant, *Ripk1^−/−^Mlkl^−/−^* and *Ripk3^−/−^* MDF cell lines were generated in-house as previously described [20,63,64]. These cell lines were derived from the tails of *Mlkl^−/−^* [20] and *Ripk3^−/−^* [65] C57BL/6 mice, with *Ripk1^−/−^* [66] and *Mlkl^−/−^* [20] mice crossed to generate the *Ripk1^−/−^Mlkl^−/−^* strain. U937 cells originate from the American Type Culture Collection (ATCC). All cells were routinely monitored for mycoplasma.

### Phenotypic screen

The phenotypic screen was performed as previously described [36,37]. Briefly, 5632 compounds from an in-house (WEHI) small molecule library and 40 well-characterised kinase inhibitors were screened against MDF cells expressing the MLKL Q343A mutant. For the 5632 compounds from the WEHI library, 384-well white clear-bottom plates (Corning) containing the compounds (10 µM) were prepared at Compounds Australia. WT MDF cells expressing MLKL Q343A were seeded at 2.0 × 10^3^ cells per well into the plates using the Nanodrop Nano-Dispensing Liquid Handler (Gilson) and incubated with the compounds for 2 h, prior to the addition of doxycycline (1 µg/ml) to induce expression of the MLKL Q343A protein. After 24 h, cell viability was determined using the CellTiter-Glo Assay (Promega) by measuring luminescence using an EnVision plate reader (PerkinElmer). For the 40 kinase inhibitors, WT and *Mlkl^−/−^* MDF cells expressing MLKL Q343A were seeded at 5.0 × 10^3^ cells per well in 96-well plates and allowed to settle for ∼6 h at 37°C 10% CO_2_. Cells were treated with the kinase inhibitors (1 µM) for 1 h and then expression of the MLKL Q343A protein was induced with doxycycline (1 µg/ml). After 18 h, cell viability was determined using the CellTiter-Glo Assay (Promega) by measuring luminescence using an EnVision plate reader (PerkinElmer). Raw luminescence data were normalised to the uninduced (no doxycycline) controls (100% cell viability) using GraphPad Prism.

### FACS cell death assays

FACS cell death assays were performed as previously described [37,48] using two protocols. In the first protocol, WT MDF cells were seeded at 1.0 × 10^5^ cells per well in 24-well plates and allowed to settle for 4 h at 37°C 10% CO_2_. Cells were treated with ABT-869 (0.2 µM, 0.6 µM, 1 µM, 5 µM), WEHI-615 (0.2 µM, 0.6 µM, 1 µM, 5 µM) or DMSO alone and Q-VD-OPh (10 µM) for 30 min before addition of TNF (100 ng/ml) and Smac mimetic (Compound A; 500 nM). After 24 h, cells were harvested, stained with 1 µg/ml propidium iodide (PI; Sigma–Aldrich) and PI positive cells were quantified by flow cytometry on a BD FACSCalibur instrument. In the second protocol, WT MDF cells were seeded at 5.0 × 10^4^ cells in 500 µl per well in 24-well plates (Falcon, cat #353047) and allowed to settle for 24 h at 37°C 10% CO_2_. Cells were treated with ABT-869 (5-point 3-fold titration from 10 µM), GSK′872 (1 µM or 10 µM), GSK′843 (1 µM or 10 µM), DMSO alone or left untreated for 1 h. Cells were then stimulated with TSQ or TSZ for 24 h to induce necroptosis, or left unstimulated. For all compounds and reagents, 0.5 µl of 1000× stocks were used. Cells were harvested, stained with 2 µg/ml propidium iodide (PI; Sigma–Aldrich) and PI positive cells were quantified by flow cytometry on a BD FACSCalibur instrument.

### IncuCyte cell death assays

IncuCyte cell death assays were performed as previously described [19,23,26]. Briefly, U937 cells were seeded at 3.5 × 10^4^ cells per well in 96-well plates. Cells were treated with ABT-869 (8-point 2-fold titration from 40 µM), WEHI-615 (8-point 2-fold titration from 40 µM), positive control compounds (2 µM NSA and 2 µM GSK′481), DMSO alone or left untreated for 1 h. Cells were then stimulated with TSQ for 48 h or TSI for 24 h to induce necroptosis, or left unstimulated. Cell death was monitored over the 24 or 48 h period by SPY505 (live cells; Spirochrome) and propidium iodide (PI; dead cells; Sigma–Aldrich) uptake using live cell imaging on an IncuCyte SX5 (Sartorius) instrument using the 10× objective and phase contrast, green and orange channel settings with scans every hour. The percentage of PI positive cells over time was quantified using the IncuCyte software. End point data were fit to the inhibitor vs response (three parameters) model of non-linear regression to obtain IC_50_ values using GraphPad Prism.

### Discoverx KINOME*scan* binding assays

Binding affinities (*K*_D_) of ABT-869 and WEHI-615 for human MLKL, RIPK1 and RIPK3 were obtained from DiscoverX KINOME*scan*, a commercial kinase inhibitor binding platform, using the *Kd*ELECT service [43]. Briefly, active/pseudoactive site competition binding assays were performed by measuring compound binding to a given kinase/pseudokinase through competition with a promiscuous probe. The ability of ABT-869 and WEHI-615 to compete with this immobilised, active-site directed ligand was quantitatively measured via qPCR of the DNA tagged-protein. ABT-869 and WEHI-615 were tested in an 11-point 3-fold dilution series against human MLKL (full-length), RIPK1 (kinase domain) and RIPK3 (kinase domain) to obtain *K*_D_ values.

### Cellular thermal shift assays

CETSA was performed as previously described [37,48]. Briefly, *Mlkl^−/−^* MDF cells were treated with doxycycline (20 ng/ml) for 4 h to induce expression of the MLKL Q343A mutant and then seeded to obtain 1.5 × 10^6^ cells per temperature point. WT MDF or U937 cells were seeded to obtain 1.5 × 10^6^ cells per temperature point. Cells were treated with DMSO or compounds (20 µM), as specified, for 1 h at 37°C 10% CO_2_. Cells were spun down, washed with 5 ml DPBS (Gibco), resuspended in DPBS containing cOmplete EDTA-free Protease Inhibitor Cocktail (Roche) and aliquoted into 100 µl samples per temperature point in PCR tubes. Live cell suspensions were heated across a temperature gradient (below) for 3 min in a T100 Thermal Cycler (Bio-Rad), cooled at room temperature for 3 min, snap frozen in liquid N_2_ and stored at −80°C overnight.

**Table:** 

Protein	Cell line	Temperature gradient (°C)
Mouse MLKL Q343A	*Mlkl^−/−^* MDF	43.5, 44.1, 45.3, 47.2, 49.4, 51.0
Mouse MLKL	MDF	45.3, 47.2, 49.4, 51.0, 52.2, 53.0
Mouse RIPK1	MDF	43.9, 45.9, 48.2, 49.9, 51.2, 52.0
Mouse RIPK3	MDF	48.9, 50.9, 53.2, 55.0, 56.2, 57.0
Human MLKL	U937	46.6, 47.7, 49.5, 51.6, 53.2, 54.3
Human RIPK1	U937	44.0, 44.7, 45.9, 47.9, 50.2, 51.9
Human RIPK3	U937	44.7, 45.9, 47.9, 50.2, 51.9, 53.2

Samples were freeze-thawed once to lyse the cells. Soluble protein was separated from precipitated protein via centrifugation at 17 000×***g*** for 30 min at 4°C. Soluble protein fractions were resolved by SDS–PAGE and analysed by Western blot. Briefly, 10 µl of each soluble protein sample was added to 10 µl of NuPAGE LDS Sample Buffer (Invitrogen) containing TCEP (Sigma–Aldrich). Samples were heated at 95°C for 10 min and then 15 µl of each sample was run on a gel along with the Precision Plus Kaleidoscope Prestained Protein Standard (Bio-Rad), using either NuPAGE 4–12% Bis-Tris 1.0 mm × 20-well Midi Gels (Invitrogen) with NuPAGE MES SDS Running Buffer (Invitrogen), or 4–15% Criterion TGX Stain-Free 26 well 15 µl Protein Gels (Bio-Rad) with Tris/Glycine Buffer (Bio-Rad). Western blots were performed according to the Western Blotting General Procedure above.

### Recombinant protein cloning, expression and purification

Recombinant kinase domains of mouse and human RIPK1 and RIPK3 were expressed and purified from *Sf*21 insect cells from bacmids generated using the Bac-to-Bac method, as detailed previously [67,68]. Bacmids were generated in DH10MultiBac *E. coli* (ATG Biosynthetics) from pFastBac HT B (Invitrogen) derived vectors encoding an in-frame TEV protease-cleavable, N-terminal His_6_ tag as before [48,69] or an in-house derivative vector encoding a non-cleavable in-frame Streptavidin Binding Peptide (SBP)-tag N-terminal to the kinase domain sequence, following the TEV protease cleavage site [9]. Bacmids were used to generate baculoviruses from *Sf*21 insect cells. Transfections were performed using Cellfectin II Reagent (Thermo Fisher), and Insect-XPRESS Media (Lonza) was used for all *Sf*21 cultures. Initially, P1 virus was generated in 6-well plates at 27°C in a humidified static incubator, and P2 virus by addition of 1 ml of P1 virus to 100 ml of 1.5 × 10^6^ cells/ml culture shaking at 27°C, 130 rpm in 1 L Schott bottles. Expressions were performed in 2.8 L Fernbach flasks by addition of 50 ml of P2 virus to 0.5 L *Sf*21 cells at 3–4 × 10^6^ cells/ml shaking at 27°C, 90 rpm for 40–48 h. Infected cells were pelleted, lysed by sonication following resuspension in 20 mM Tris pH 8.0, 500 mM NaCl, 20% v/v glycerol, 15 mM imidazole, 0.5 mM TCEP buffer containing cOmplete EDTA-free Protease Inhibitor Cocktail (Roche), and cellular debris pelleted at 45 000×***g***. Supernatant was subjected to immobilised metal affinity chromatography using cOmplete His-Tag Purification Resin (Roche) and removal of the His_6_ tag by TEV protease treatment, followed by dialysis, further Ni^2+^-chromatography to remove the TEV protease and uncut material, before size exclusion chromatography using a Superdex 200 column (GE Healthcare) with elution in 20 mM HEPES pH 7.5, 200 mM NaCl, 5% v/v glycerol, as per established procedures [9,48,50,68–70]. All purification steps were performed at 4°C. Size exclusion chromatography fractions containing purified protein were ascertained using reducing Stain-Free SDS–PAGE (Bio-Rad) and pure fractions were pooled and concentrated by centrifugal ultrafiltration at 4000×***g*** to 1–5 mg/ml, as assessed by absorbance at 280 nm (A_280_), before aliquots were snap frozen in liquid N_2_ for storage at −80°C until use. Mouse SBP-RIPK1 (2–372), mouse SBP-RIPK3 (3–353), human SBP-RIPK1 (2–373) and human RIPK3 (2–316; C3S, C110A) were used in Thermal Shift Assays (TSA); and mouse SBP-RIPK1 (2–372), mouse SBP-RIPK3 (3–353), human RIPK1 (2–373) and human RIPK3 (2–311) in kinase activity assays.

### *In vitro* thermal shift assays

TSA was performed as previously described [20,69–71]. Briefly, ABT-869 (7-point 3-fold titration from 30 µM), WEHI-615 (7-point 3-fold titration from 30 µM) and positive control (mouse RIPK1: 30 µM Compound 2 [48]; mouse RIPK3: 30 µM GSK′872; human RIPK1: 30 µM GSK′481; human RIPK3: 30 µM GSK′872) compound solutions were prepared. Compounds, DMSO or buffer were added to each protein (mouse RIPK1: 9.5 µg; mouse RIPK3: 10 µg; human RIPK1: 12 µg; human RIPK3: 6.5 µg) in buffer (20 mM HEPES pH 7.5, 200 mM NaCl, 5% v/v glycerol) in 0.1 ml strip tubes with caps (Gene Target Solutions) on ice. SYPRO Orange (Sigma–Aldrich) was diluted 1 : 20 with DMSO and then 1 µl was added to the tubes on ice. The total volume was 25 µl containing 8% v/v DMSO, aside from the buffer only (apo) controls that contained 4% v/v DMSO. Assays were performed using a Rotor-Gene Q instrument (Qiagen) where the temperature was increased by 1°C per minute from 25°C to 80°C and the fluorescence at each time increment was detected at 555 nm. Raw fluorescence data were truncated after the fluorescence maxima, normalised and fit to the Boltzmann sigmoidal model of non-linear regression to obtain melting temperature (*T*_M_) values using GraphPad Prism. Δ*T*_M_ values were then calculated by subtracting *T*_M_ DMSO from *T*_M_ compound and fit to the one site specific binding model of non-linear regression to obtain *K*_D_ values using GraphPad Prism.

### Kinase activity assays

Human RIPK1 and RIPK3 kinase assays were performed as previously described [9,37] using the ADP-Glo Kinase Assay (Promega) with a 10-point 3-fold titration of ABT-869 or WEHI-615 from 50 µM (human RIPK1) or 100 µM (human RIPK3). Mouse RIPK1 and RIPK3 kinase assays were performed in 96-well plates (Falcon, cat #353296) using the ADP-Glo Kinase Assay (Promega). ABT-869 (9-point 3-fold titration from 100 µM), WEHI-615 (9-point 3-fold titration from 100 µM) and positive control (mouse RIPK1: 1 µM Compound 2 [48]; mouse RIPK3: 1 µM GSK′872) compound solutions were prepared. Compounds or DMSO were incubated with mouse RIPK1 (200 nM) or mouse RIPK3 (10 nM) in buffer (50 mM Tris pH 7.5, 50 mM NaCl, 30 mM MgCl_2_, 0.05% w/v BSA, 0.01% v/v Triton X-100, 1 mM DTT) for 30 min at room temperature. ATP (10 µM) was added and the kinase reaction was incubated for 4 h at room temperature in a reaction volume of 25 µl containing 1% v/v DMSO. An amount of 25 µl ADP-Glo Reagent (Promega) was added and incubated for 1 h at room temperature. An amount of 50 µl Kinase Detection Reagent (Promega) was added and incubated for 1 h at room temperature. Luminescence was measured on a CLARIOstar plate reader (BMG Labtech). Raw luminescence data were normalised to the negative control (DMSO; 0% inhibition) and the positive control (mouse RIPK1: 1 µM Compound 2; mouse RIPK3: 1 µM GSK′872; 100% inhibition), then fit to the inhibitor vs response (three parameters) model of non-linear regression to obtain IC_50_ values using GraphPad Prism.

### Phosphorylation time course

WT, *Ripk1^−/−^Mlkl^−/−^* and *Ripk3^−/−^* MDF cell lines were seeded at 5.0 × 10^5^ cells in 3 ml per well in 6-well plates (Corning, cat #3516) and allowed to settle for 24 h at 37°C 10% CO_2_. WT cells were stimulated with TSI for 1, 2, 3, or 4 h, or left unstimulated, to induce phosphorylation of RIPK1 and RIPK3. *Ripk1^−/−^Mlkl^−/−^* and *Ripk3^−/−^* cells were left unstimulated. Cells were harvested, washed with 1 ml DPBS (Gibco) and lysed in 30 µl of ice-cold RIPA buffer (25 mM Tris–HCl pH 7.5, 150 mM NaCl, 1% v/v Nonidet P-40, 1% w/v Na Deoxycholate, 0.1% w/v SDS) containing cOmplete EDTA-free Protease Inhibitor Cocktail (Roche), PhosSTOP Phosphatase Inhibitor Cocktail (Roche) and benzonase nuclease (Sigma–Aldrich) for 30 min on ice. An amount of 30 µl of NuPAGE LDS Sample Buffer (Invitrogen) containing TCEP (Sigma–Aldrich) was added to the whole cell lysates, which were then stored at −20°C until SDS–PAGE and Western blots were performed. Briefly, samples were thawed at room temperature, heated at 95°C for 10 min and 10 µl of each sample was run on NuPAGE 4–12% Bis-Tris 1.5 mm × 10-well Mini Gels (Invitrogen) using NuPAGE MES SDS Running Buffer (Invitrogen) along with the Precision Plus Kaleidoscope Prestained Protein Standard (Bio-Rad). Western blots were performed according to the Western Blotting General Procedure above.

### Phosphorylation assays

WT, *Ripk1^−/−^Mlkl^−/−^* and *Ripk3^−/−^* MDF cell lines were seeded at 3.0 × 10^5^ cells in 1 ml per well in 12-well plates (Corning, cat #3512) and allowed to settle for 24 h at 37°C 10% CO_2_. WT cells were treated with DMSO alone or compounds (1 µM or 10 µM), as specified, for 2 h. *Ripk1^−/−^Mlkl^−/−^* and *Ripk3^−/−^* cells were left untreated. Cells were stimulated with TSI to induce phosphorylation of RIPK1 and RIPK3, or left unstimulated, for 2 h. For all compounds and reagents, 1 µl of 1000× stocks were used. Cells were harvested, washed with 500 µl DPBS (Gibco) and lysed in 25 µl of ice-cold RIPA buffer (25 mM Tris–HCl pH 7.5, 150 mM NaCl, 1% v/v Nonidet P-40, 1% w/v Na Deoxycholate, 0.1% w/v SDS) containing cOmplete EDTA-free Protease Inhibitor Cocktail (Roche), PhosSTOP Phosphatase Inhibitor Cocktail (Roche) and benzonase nuclease (Sigma–Aldrich) for 30 min on ice. Whole cell lysates were stored at −80°C until protein quantification, SDS–PAGE and Western blots were performed. Briefly, whole cell lysates were thawed on ice then quantified using the BCA Protein Assay with bicinchoninic acid solution (Sigma–Aldrich) and copper sulfate solution (Sigma–Aldrich). NuPAGE LDS Sample Buffer (Invitrogen) containing TCEP (Sigma–Aldrich) was added to the whole cell lysates to obtain 20 µg protein in 20 µl total volume per sample. Samples were heated at 95°C for 10 minutes and 20 µl of each sample was run on NuPAGE 4–12% Bis-Tris 1.0 mm × 12-well Mini Gels (Invitrogen) using NuPAGE MES SDS Running Buffer (Invitrogen) along with the Precision Plus Kaleidoscope Prestained Protein Standard (Bio-Rad). Western blots were performed according to the Western Blotting General Procedure above.

## Data Availability

All data and reagents are available from the authors upon request. Uncropped Western blots are included as supplementary data.

## Open Access

Open access for this article was enabled by the participation of the University of Melbourne in an all-inclusive *Read & Publish* agreement with Portland Press and the Biochemical Society under a transformative agreement with CAUL.

## Abbreviations

CETSA: cellular thermal shift assays
Dox: doxycycline
IC50: half maximal inhibitory concentration
MDF: mouse dermal fibroblast
MLKL: mixed lineage kinase domain-like
PI: propidium iodide
RIPK1: Receptor interacting protein kinase 1
RIPK3: Receptor interacting protein kinase 3
SBP: streptavidin binding peptide
*T*_M_: melting temperature
TNF: tumour necrosis factor
TSA: thermal shift assays
TSI: TNF, Smac-mimetic, IDN-6556
TSQ, TNF: Smac-mimetic, QVD-OPh
TSZ, TNF, Smac-mimetic: zVAD-fmk
UT: untreated
WT: Wild-type
